# Pyruvate-Driven Oxidative Phosphorylation is Downregulated in Sepsis-Induced Cardiomyopathy: A Study of Mitochondrial Proteome

**DOI:** 10.1097/SHK.0000000000001858

**Published:** 2021-09-09

**Authors:** Briana K. Shimada, Liron Boyman, Weiliang Huang, Jing Zhu, Yang Yang, Fengqian Chen, Maureen A. Kane, Nagendra Yadava, Lin Zou, W. Jonathan Lederer, Brian M. Polster, Wei Chao

**Affiliations:** ∗ Translational Research Program, Department of Anesthesiology and Center for Shock, Trauma and Anesthesiology Research, Baltimore, Maryland; † The Department of Physiology and Center for Biomedical Engineering and Technology, University of Maryland School of Medicine, Baltimore, Maryland; ‡ Department of Pharmaceutical Sciences, University of Maryland School of Pharmacy, Baltimore, Maryland

**Keywords:** Bioenergetics, cardiomyopathy, citric acid cycle, mitochondria, oxidative phosphorylation, proteomics, sepsis

## Abstract

**Background:**

Sepsis-induced cardiomyopathy (SIC) is a major contributing factor for morbidity and mortality in sepsis. Accumulative evidence has suggested that cardiac mitochondrial oxidative phosphorylation is attenuated in sepsis, but the underlying molecular mechanisms remain incompletely understood.

**Methods:**

Adult male mice of 9 to 12 weeks old were subjected to sham or cecal ligation and puncture procedure. Echocardiography *in vivo* and Langendorff-perfused hearts were used to assess cardiac function 24 h after the procedures. Unbiased proteomics analysis was performed to profile mitochondrial proteins in the hearts of both sham and SIC mice. Seahorse respirator technology was used to evaluate oxygen consumption in purified mitochondria.

**Results:**

Of the 665 mitochondrial proteins identified in the proteomics assay, 35 were altered in septic mice. The mitochondrial remodeling involved various energy metabolism pathways including subunits of the electron transport chain, fatty acid catabolism, and carbohydrate oxidative metabolism. We also identified a significant increase of pyruvate dehydrogenase (PDH) kinase 4 (PDK4) and inhibition of PDH activity in septic hearts. Furthermore, compared to sham mice, mitochondrial oxygen consumption of septic mice was significantly reduced when pyruvate was provided as a substrate. However, it was unchanged when PDH was bypassed by directly supplying the Complex I substrate NADH, or by using the Complex II substrate succinate, or using Complex IV substrate, or by providing the beta-oxidation substrate palmitoylcarnitine, neither of which require PDH for mitochondrial oxygen consumption.

**Conclusions:**

These data demonstrate a broad mitochondrial protein remodeling, PDH inactivation and impaired pyruvate-fueled oxidative phosphorylation during SIC, and provide a molecular framework for further exploration.

## INTRODUCTION

Sepsis-induced cardiomyopathy (SIC) is a common complication of sepsis that results in circulatory failure and high mortality (1–4). While the etiology of SIC is complex and multifactorial, several studies have provided evidence that mitochondrial dysfunction plays a key role (3, 5). Clinically, there is a possible association between the degree of mitochondrial dysfunction, disease severity, and septic patient outcomes (3, 6). Yet, the molecular mechanisms underlying the mitochondrial dysfunction in the septic heart remain incompletely understood.

The mitochondria are the primary source of ATP to the working heart and encompass roughly 30 percent of the total volume of a heart muscle cell, the highest volume fraction of all mammalian cell types (7). Daily energy consumption by the heart is the highest of all the organs (8), requiring a constant and tightly regulated mitochondrial ATP production to meet these high energy demands (9, 10). Mitochondrial oxidative phosphorylation in the heart is fueled by fatty acid oxidation with additional contribution from the end product of carbohydrate (e.g., glucose) catabolism, pyruvate. Fatty acids are metabolized by beta-oxidation whereas pyruvate is decarboxylated to form acetyl-CoA by pyruvate dehydrogenase (PDH), the enzyme that links cytoplasmic glycolysis metabolic pathway to the mitochondrial citric acid cycle. The metabolism of fatty acids and pyruvate inside the mitochondrial matrix yields reduced nicotinamide adenine dinucleotide (NADH) and reduced flavin adenine dinucleotide (FADH_2_). NADH and FADH_2_ are the source of energy powering protons pumping out of the mitochondrial matrix by the electron transport chain (ETC), which generate a large electrochemical proton gradient across the mitochondrial inner membrane. This gradient drives ATP production by ATP synthase (complex V) (11). The synthesized ATP then exits the mitochondrial matrix via adenine nucleotide translocase (ANT) and is used to fuel the production of contractile force, calcium signaling, and maintaining ionic gradients across sarcolemmal and organellar membranes.

A number of studies demonstrate altered mitochondrial energy production and abnormal oxidative phosphorylation in the hearts of septic mice or mice treated with proinflammatory agents (3). These include decreases in oxygen consumption, decreased mitochondrial ATP synthesis due to impaired activities of several ETC complexes (12–15), and reduced adenine nucleotide translocator 1 (ANT1) (16). While these reports highlight the possible role of mitochondrial dysfunction in SIC, others report enhanced mitochondrial function in sepsis (17). Importantly, a recent study shows that restoring PDH activity in sepsis promotes mitochondrial bioenergetics in the splenocytes and hepatocytes and improves sepsis survival in mice (18).

In this study, we employed an unbiased proteomics approach to profile the mitochondrial protein expression in the heart. Among 665 detected proteins with a known mitochondrial localization or mitochondrial association, 35 varied significantly between healthy and septic hearts. These included those with key functions in glucose and fatty acid metabolism, as well as subunits of the electron transport chain. Of particular interest, we found an increase in PDH kinase isozyme 4 (PDK4), whose function is to phosphorylate pyruvate dehydrogenase (PDH) and inhibit its function (19, 20), and marked PDH inactivation in the septic hearts. Moreover, employing Seahorse respirator assay, we found that mitochondrial oxygen consumption in the septic hearts was significantly reduced when pyruvate was provided as fuel. These findings support the hypothesis that mitochondrial protein remodeling such as PDK4 upregulation and PDH phosphorylation/inactivation may play an important role in cardiac metabolic dysfunction during SIC.

## METHODS

### Animals

C57BL/6 mice were purchased from Jackson Laboratory (Bar Harbor, ME). Adult male mice were between 9 and 12 weeks of age and weighed between 20 and 30 g. The animal protocols were approved by the Institutional Animal Care and Use Committee, University of Maryland School of Medicine (Approval Number: 0918001, Baltimore, MD). All animal experiments were performed in compliance with the relevant guidelines and regulations of the National Institutes of Health (Bethesda, MD) and the ARRIVE guidelines of the National Centre for the Replacement, Refinement, and Reduction of Animals in Research (London, EN).

### Cecal ligation and puncture

A clinically relevant mouse model of sepsis was generated by cecal ligation and puncture (CLP) surgery as described previously with minor modifications (21–24). Briefly, mice were anesthetized with ketamine (100 mg/kg) and xylazine (10 mg/kg). The abdominal cavity was opened in layers and the feces were gently migrated to fill the distal part of the cecum. The cecum was ligated approximately 1.0 cm from the tip and was punctured with an 18-gauge needle. A small droplet of feces was extruded to ensure patency of the puncture site and the cecum was returned to the abdominal cavity. Sham-operated mice underwent laparotomy without CLP. The abdominal wall was closed in layers with 7–0 sterile sutures. After surgery, prewarmed saline (50 mL/kg body weight) was administered subcutaneously to both sham and CLP mice. Bupivacaine (3 mg/kg) and buprenorphine (0.1 mg/kg) were given postoperatively to manage pain.

### Echocardiography

Echocardiography was performed in non-anesthetized mice as previously described (21).

### Langendorff measurements

Mice were heparinized (1000 IU/kg, intraperitoneal) and anesthetized with ketamine (100 mg/kg) and xylazine (10 mg/kg). Following confirmation of anesthesia, hearts were then excised and subjected to an *ex vivo* Langendorff perfusion model as previously described with minor modifications (21). A water-filled balloon was introduced into the left ventricle to record LV pressure (PowerLab, AD Instruments, Denver, CO). Hearts were paced at 420 bpm and left ventricular developed pressure (LVDP, left ventricular end systolic pressure—left ventricular end diastolic pressure, LVESP–LVEDP) was recorded continuously for 30 min. LVDP was taken as an average of the pressure over those 30 min.

### ADP/ATP and ATP assay

For the ADP/ATP ratio assay, hearts were snap-frozen and homogenized in ice-cold 2N perchloric acid (PCA) at 100 μL/10 mg tissue using a tissue tear for 5 s followed with Dounce homogenization for 25 to 30 passes. Samples were kept on ice for 45 min and centrifuged at 13,000 × *g* for 2 min at 4°C. One hundred microliters of supernatant was diluted with ATP assay buffer (Abcam, Cambridge, MA) and neutralized with 2 M KOH. Samples were then centrifuged again at 13,000 × *g* for 15 min at 4°C and supernatant was collected. Deproteinized and neutralized samples were then run using a commercially available ADP/ATP ratio assay kit from Abcam (Catalog No: ab65313) per manufacturer's instructions. ADP/ATP ratio were calculated as described in the manufacturer's protocol. For the ATP assay, mice hearts were snap frozen and homogenized in ice-cold 1% trichloroacetic acid and kept on ice for 45 min and centrifuged. Supernatants were neutralized with 1 M KOH. Deproteinized and neutralized samples were then assayed for ATP content as previously described (10).

### Isolation of cardiac mitochondria

Mouse cardiac mitochondria were isolated as we previously described (10). It is worth noting that, in our protocol, we took additional steps that are not routinely used to increase purity but with the cost of lower yield. We pelleted the nonmitochondrial fractions at low-speed centrifugation of 600 × *g* three times. To pellet and isolate mitochondria, we centrifuged our samples at 3,200 × *g* instead of the more commonly used 10,000 × *g*. In addition, after we obtained the mitochondrial pellet, we repeated this step three more times to further discard any possible cytosolic or other soluble materials of the supernatant, whereas typical isolation protocols utilize only one cycle. As result, the mitochondrial preparations contain only trace amounts of GAPDH and SERCA (Suppl. Fig. 1), two proteins highly expressed in the cytoplasm and the sarcoplasmic reticulum, respectively, suggesting that the cardiac mitochondrial preparations in our study were highly pure and with minimal cytosolic or organelle contaminations. Subsequently, a BCA assay was used to quantify the concentration of isolated mitochondria (in mg mitochondrial protein per mL). The mitochondria were kept on ice and used for respiration measurements within 4 h of isolation. The remaining mitochondria were stored at −80°C for Western blots. Mitochondrial assay solution (MAS, 1X) was comprised of: 70 mM sucrose, 220 mM mannitol, 10 mM KH_2_PO_4_, 5 mM MgCL_2_, 2 mM HEPES, 1 mM EGTA, 0.2% (w/v) fatty acid-free BSA (pH = 7.2) (25). Drugs and substrates were prepared as described previously and diluted to the following final concentrations: 4 mM ADP, 2.5 μg/mL oligomycin, 4 μM FCCP, 4 μM antimycin A, 10 mM succinate, 2 mM malate, 2 μM rotenone, and 10 mM pyruvate (25).

### Seahorse respirator assay

All Seahorse assays were performed using an XF24 plate as described previously with minor modifications (25). Based on the type of substrates used for the respiration assay, various amounts of isolated cardiac mitochondria in a volume of 50 μL, that is, 2.5 μg (succinate and rotenone), 5 μg (pyruvate and malate), or 7.5 μg (palmitoylcarnitine and malate) were delivered to each well in triplicate or quadruplicate technical replicates. Mitochondria were then centrifuged at 2000 × *g* for 20 min at 4°C in a swinging bucket microplate adapter to attach the mitochondria to the plate. After centrifugation, 625 μL of warm 1X MAS + substrate was added to each well. Either the complex I-linked substrates pyruvate (10 mM) and malate (2 mM), the complex II substrate succinate (10 mM) in the presence of the Complex I inhibitor rotenone (2.5 μM), or the beta-oxidation substrate palmitoylcarntine (40 μM) and malate (1 mM) were given to the mitochondria in the 1X MAS. The plate was then allowed to equilibrate at 37°C for 10 min and subsequently transferred to the XF24 instrument for oxygen consumption rate (OCR) measurements. Three basal measurements were recorded for 3 min each with 1 min of mixing between each recording. Immediately following the last basal measurement, the following injection protocol was used:

1. Port A: 4 mM ADP
2. Port B: 2.5 μg/mL oligomycin
3. Port C: 4 μM FCCP
4. Port D: 4 μM antimycin A (AA).

Mix and measure times were 1 and 3 min, respectively, and drugs were injected immediately following the previous measurement. For the NADH-based respiration assay in which Complex IV activity was also specifically determined (26–28), 0.156 μg of isolated mitochondria were delivered to each well in triplicate or quadruplicate technical replicates. Three basal measurements were recorded for 2 min each with 2 min of mixing between each recording. Isolated mitochondria were then subjected to the following injection protocol:

1. Port A: 1 mM NADH, 40 μg/mL alamethicin, and 100 μM cytochrome C.
2. Port B: 2 μM rotenone.
3. Port C: 0.5 mM TMPD, 2 mM ascorbate, and 4 μM antimycin A.
4. Port D: 50 mM azide.

Wait, mix, and measure times were all 2 min long and drugs were injected immediately following the previous measurement.

### Proteomics

Mitochondria isolated from the hearts of sham or CLP mice were solubilized in 5% sodium deoxycholate and were washed, reduced, alkylated and trypsinolyzed in a filter as previously described (29, 30). Tryptic peptides were separated on a nanoACQUITY UPLC analytical column (BEH130 C18, 1.7 μm, 75 μm × 200 mm, Waters Corporation) over a 165-min linear acetonitrile gradient (3–40%) with 0.1% formic acid on a Waters nano-ACQUITY UPLC system and analyzed on a coupled Thermo Scientific Orbitrap Fusion Lumos Tribrid mass spectrometer as described (31). Full scans were acquired at a resolution of 240,000, and precursors were selected for fragmentation by collision-induced dissociation (normalized collision energy at 35%) for a maximum 3-s cycle. Tandem mass spectra were searched against a UniProt mouse reference proteome using Sequest HT algorithm (32) and MS Amanda algorithm (33) with a maximum precursor mass error tolerance of 10 ppm. Carbamidomethylation of cysteine and deamidation of asparagine and glutamine were treated as static and dynamic modifications, respectively. Resulting hits were validated at a maximum false discovery rate of 0.01 using a semi-supervised machine learning algorithm Percolator (34). Label-free quantifications were performed using Minora, an aligned AMRT (Accurate Mass and Retention Time) cluster quantification algorithm (Thermo Scientific, 2017). Protein abundance ratios between the CLP and the sham were measured by comparing the MS1 peak volumes of peptide ions, whose identities were confirmed by MS2 sequencing as described above.

### RNA extraction and qRT-PCR

Myocardial tissue RNA was extracted using TRIzol (Sigma) and quantified using the Nanodrop as reported previously (23). The sequences of the primers used in the study are listed in Table 2.

### Western blot

Whole hearts were washed in PBS and snap-frozen in liquid nitrogen. Isolated mitochondria were stored at −80°C. Both tissue and isolated mitochondria were then resuspended in NP-40 lysis buffer containing complete protease inhibitor cocktails (Roche Diagnostics, Indianapolis, IN) and protein concentrations were quantified using a BCA protein assay. Equivalent protein concentrations were fractionated by SDS-PAGE under reducing conditions and blotted with the following antibodies: PDK4 (Proteintech, Rosemont, IL, 1:1000), cytochrome C (BD, Franklin Lakes, NJ, 1:5000), SERCA2 (Santa Cruz, Dallas, TX, 1:200), GAPDH (CST, Danvers, MA 1:1000), p-PDHE1α^serine293^ (Abcam, Watham, MA, 1:1000) and PDH antibody cocktail (Abcam, 1:1000).

### PDH activity assay

PDH activity was assessed using a commercially available PDH activity kit from Abcam (Catalog No: ab109902). In the assay, the PDH enzyme was immunocaptured within the wells of the microplate and activity was determined by following the reduction of NAD+ to NADH, coupled to the reduction of a reporter dye to yield a colored reaction product with an increase in absorbance at 450 nm at room temperature. Hearts were homogenized in ice-cold PBS containing protease and phosphatase inhibitors. Protein concentration was determined using a BCA protein assay and each sample was adjusted to 200 μg of protein. The assay was then performed to the manufacturer's specifications in kinetic mode for 15 min at room temperature. The rate (ΔmOD/min) was then calculated by using the following formula: $\frac{Rate=(Absorbance_{t2}-Absorbancel_{t1})}{time(\min utes)}$.

### Statistical analysis

Statistical analysis was performed using GraphPad Prism 8 software (GraphPad, La Jolla, CA). Unless stated otherwise, the distributions of the continuous variables were expressed as the mean ± SEM. Proteomic comparisons between the CLP mice and the sham were performed by FDR corrected, one-way ANOVA test and *z*-score. D’Agostino and Pearson (single comparison) or Kruskal–Wallis (multiple comparisons) normality tests were used to evaluate the normality of the data. If normal, the statistical significance of the difference between groups was measured by *t* test or one-way ANOVA with Tukey's post hoc test or two-way ANOVA with Sidak's post hoc test for more than two groups. If the data did not pass the normality test, nonparametric tests (Mann–Whitney for single comparisons) were used to derive the *P* values. The null hypothesis was rejected for *P* < 0.05 with the two-tailed *t* test.

## RESULTS

### Polymicrobial sepsis induces significant cardiac dysfunction and energy deficit

**Fig. 1:**
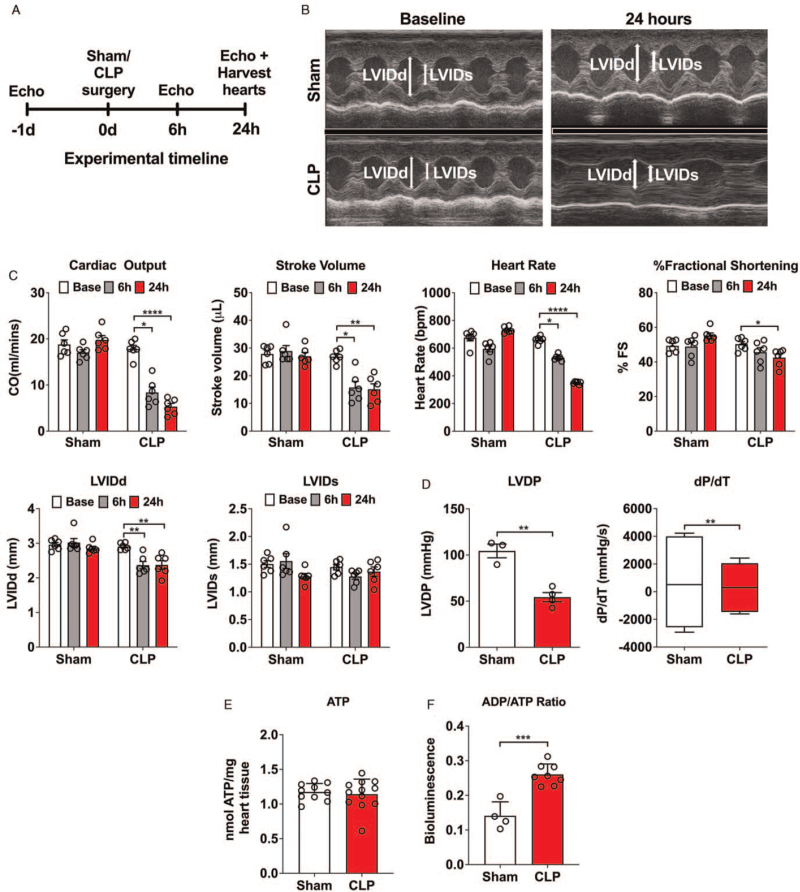
Cardiac dysfunction and ATP and ADP/ATP ratio in sepsis mice. **(**A) The timeline of experiments. A baseline echocardiography (Echo) was performed one day prior to surgical procedures. The next day, sham (laparotomy only) or CLP (laparotomy plus cecal ligation and puncture) surgery was performed and Echo used to assess cardiac function at 6 and 24 h postsurgery. After the 24 h Echo, animals were sacrificed, and mitochondria were isolated for subsequent experiments. (B) Representative Echo M-mode images showing LVIDd/LVIDs at baseline and 24 h after sham or CLP procedure. (C) Echo measurements at baseline and at 6 and 24 h after sham or CLP surgery. n = 6/group. (D) *Ex vivo* Langendorff-perfused hearts. Left ventricular (LV) developed pressure (LVDP) and rate of LVDP (dP/dT) were measured at 24 h after sham and CLP procedures. n = 3 to 4/group. (E–F) Total ATP and ADP/ATP ratio were measured 24 h after sham and CLP surgery in whole hearts. (E) Total ATP, n = 10 (sham) and 12 (CLP). (F) ADP/ATP ratio. n = 4 (sham) and 8 (CLP). Each error bar represents mean ± SEM. Data in (C) were analyzed using 2-way ANOVA with Sidak's multiple comparisons test. Panels in (D–F) were analyzed using two-tailed *t* test. ^∗^*P* < 0.05, ^∗∗^*P* < 0.01, ^∗∗∗^*P* < 0.001.

To model sepsis-induced cardiomyopathy (SIC), we subjected mice to cecal ligation and puncture (CLP) or sham laparotomy. Cardiac function was assessed at baseline prior to surgery and again at 6- and 24-h postsurgery using echocardiography (Fig. 1A and B). We observed significantly decreased cardiac output, stroke volume, heart rate, LVIDd, and percent fractional shortening in the mice that underwent CLP surgery compared with sham (Fig. 1C). To rule out possible *in vivo* confounders associated with the load-dependent echocardiographic measurements, we isolated hearts from sham and CLP mice 24 h after procedures and subjected the mouse hearts to *ex vivo* Langendorff apparatus. Analysis of these hearts revealed a significant decrease in left ventricular developed pressure (LVDP) in the CLP group compared with sham (104.4 ± 12.9 vs. 54.3 ± 9.9 mmHg, sham vs. CLP, n = 3–4, *P* < 0.01) as well as in dP/dt maximum (3852 ± 416 vs. 2010 ± 341 mmHg/s) and minimum (−2,591 ± 293 vs. −1,376 ± 241 mmHg/s, sham vs. CLP n = 3–4, *P* < 0.01, Fig. 1D). To test if an energy deficit is present in the heart, we examined total ATP levels and the ADP/ATP ratio from sham and CLP hearts using commercially available assay kits. Interestingly, while total ATP levels remained the same between sham and CLP, CLP hearts exhibited a significantly higher ADP/ATP ratio (0.14 ± 0.02 vs. 0.26 ± 0.01, sham vs. CLP, *P* < 0.001) (Fig. 1E and F). These data indicate that CLP mice developed intrinsic cardiomyopathy and might possess myocardial energy deficit.

### Proteomics analysis reveals altered mitochondrial metabolic proteins in septic heart

**Fig. 2:**
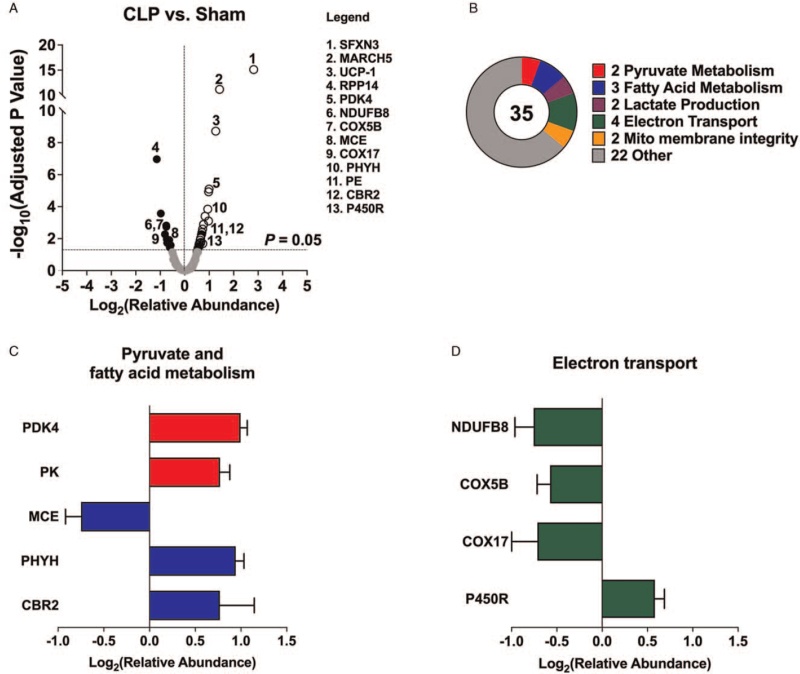
Proteomic analysis of isolated cardiac mitochondria from septic mice. Mitochondria were isolated from the hearts of sham- and CLP-operated mice and proteomic analysis was conducted via liquid chromatography-tandem mass spectrometry. (A) Volcano plot of all mitochondrial or metabolically coupled proteins found in the isolated mitochondria. All proteins detected by the mass spectrometry were referenced against a mouse reference proteome using a Sequest HT algorithm and MS Amanda algorithm as described in *Methods.* Proteins considered to be nonmitochondrial or nonmetabolically related based on their identity were excluded from the plot. Proteins with *P*-values less than 0.05 were considered significant changes between the two groups. Black dots represent decreased expression while white open dots indicate increased expression. The main proteins with differential expression are labeled in the graph legend. (B) Proteins, total 35 differentially expressed in sepsis mitochondria, are grouped in different colors based on their roles in glycolysis, fatty acid metabolism, electron transport, or mitochondria membrane integrity. (C–D) List of mitochondrial specific proteins with altered expression that are involved in either pyruvate metabolism, fatty acid metabolism, or electron transport. Each color is corresponding to different metabolic pathway as shown in (B). Each mouse heart was assessed individually by the proteomics and was not mixed together. n = 6 mitochondrial preparations from each group were used for proteomic analysis. Statistical analysis was done as described in the methods section. (CBR2, carbonyl reductase [NADPH] 2; COX 17, cytochrome c oxidase copper chaperone; COX5B, cytochrome c oxidase 5B; MARCH5, E3 ubiquitin-protein ligase; MCE, methylmalonyl-CoA epimerase; NDUFB8, NADH ubiquinone oxioreductase subunit B8; P450R, NADPH-cytochrome P450 reductase; PDK4, pyruvate dehydrogenase kinase 4; PHYH, Phytanoyl-CoA 2-Hydroxylase; PK, pyruvate kinase; SFXN13, sideroflexin-3; MARCH5; RPP14, ribonuclease P 14 subunit); UCP-1, mitochondrial brown fat uncoupling protein-1.

One of the possible molecular mechanisms behind the contractile dysfunction during sepsis is the impairment of mitochondrial bioenergetics. We hypothesize that bioenergetic impairment and subsequent myocardial dysfunction during sepsis is associated with altered mitochondrial protein expression. To test this, cardiac mitochondria were isolated from sham mice and CLP mice exhibiting clear cardiac dysfunction (n = 6/group) and evaluated using liquid chromatography-tandem mass spectrometry. Of the 665 proteins with an established physical or functional mitochondrial association that were identified in the proteomics assay, 35 were found to be significantly altered between sham and CLP mitochondria (Fig. 2A). A complete list of the 35 proteins can be found in Table 1. Notably, these 35 differentially expressed mitochondria-associated proteins are reportedly involved in various bioenergetic functions such as pyruvate metabolism, fatty acid metabolism, lactate production, electron transport, and mitochondrial membrane integrity (Fig. 2B, Table 2). Pyruvate dehydrogenase kinase 4 (PDK4), a primary regulator of PDH, was significantly up-regulated in the mitochondrial fraction isolated from septic hearts. Pyruvate kinase (PK), the enzyme responsible for catalyzing the transfer of a phosphate group from phosphoenolpyruvate (PEP) to adenosine diphosphate (ADP) to yield pyruvate and one molecule of ATP in the last step of glycolysis, was also upregulated. Meanwhile, methylmalonyl-CoA epimerase (MCE), an enzyme involved in odd-chain fatty acid catabolism, was significantly down-regulated in sepsis heart mitochondria (Fig. 2C). In addition, we discovered up-regulation of two other proteins indirectly related to fatty acid metabolism, phytanol-CoA dioxygenase (PHYH), and carbonyl reductase [NADPH] 2 (CBR2). PHYH catalyzes the first step in the alpha-oxidation of phytanic acid while CBR2 metabolizes aldehydes and ketones derived from lipid peroxidation (Fig. 2C). Moreover, a subset of electron transport chain proteins, including NADH dehydrogenase [ubiquinone] 1 beta subcomplex subunit 8 (NDUFB8, a subunit of Complex I), cytochrome c oxidase 5B (COX5B, a subunit of Complex IV), cytochrome c oxidase copper chaperone (COX17, a nuclear-encoded protein essential for the assembly of Complex IV), were downregulated, while NADPH-cytochrome P450 reductase (P450R, an enzyme required for the transfer of electrons from NADPH to cytochrome P450), was up-regulated in the mitochondria of septic mice (Fig. 2D).

**Table 1:** Proteomics analysis of cardiac mitochondrial proteins: 35 differentially expressed proteins between CLP and Sham mice

*#*		Accession	Gene name	Expression ratio (CLP/Sham)
Pyruvate metabolism				
1	Pyruvate kinase	P52480	PKM	1.706
2	Pyruvate dehydrogenase (acetyl-transferring)] kinase isozyme 4	O70571	PDK4	1.993
Fatty acid metabolism				
3	Methylmalonyl-CoA epimerase	Q9D1I5	MCEE	0.595
4	Carbonyl reductase [NADPH] 2	P08074	CBR2	1.704
5	Phytanoyl-CoA dioxygenase, peroxisomal	O35386	PHYH	1.921
Lactate production				
6	L-lactate dehydrogenase B chain	P16125	LDHB	1.481
7	L-lactate dehydrogenase	A0A1B0GSX0	LDHA	1.668
Electron transport				
8	NADH dehydrogenase [ubiquinone] 1 beta subcomplex subunit 8, mitochondrial	Q9D6J5	NDUFB8	0.592
9	Cytochrome c oxidase subunit 5B	Q9D881	GM11273	0.671
10	Cytochrome c oxidase copper chaperone	P56394	COX17	0.609
11	NADPH–cytochrome P450 reductase	P37040	P450R	1.497
Mitochondrial membrane integrity				
12	MICOS complex subunit MIC10	Q7TNS2	MINOS1	0.508
13	E3 ubiquitin-protein ligase MARCH5	Q3KNM2	MARCH5	2.685
Other				
14	Glyceraldehyde-3-phosphate dehydrogenase	A0A0A0MQF6	GAPDH	1.660
15	Cathepsin D	P18242	CTSD	1.607
16	Mitochondrial brown fat uncoupling protein 1	P12242	UCP-1	2.410
17	Phosphate carrier protein	Q8VEM8	SLC25A3	1.575
18	Cathepsin B	P10605	CTSB	1.777
19	Sideroflexin-3	Q91V61	SFXN3	7.061
20	Keratin, type II cytoskeletal 5	Q922U2	KRT5	2.413
21	B-cell receptor-associated protein 31	Q61335	BCAP31	1.631
22	39S ribosomal protein L15	Q9CPR5	MRPL15	1.571
23	Transforming protein RhoA	Q9QUI0	RHOA	1.964
24	Transport and Golgi organization 2 homolog	P54797	TANGO2	1.514
25	Fructose-bisphosphate aldolase	A6ZI44	ALDOA	1.549
26	Tripeptidyl-peptidase 1	O89023	TPP1	1.457
27	Mitochondrial thiamine pyrophosphate carrier	Q9DAM5	SLC25A19	0.645
28	Solute carrier family 35 member F6	Q8VE96	SLC35F6	1.577
29	Ribonuclease P 14 subunit (Human)	J3QMX0	RPP14	0.453
30	Coiled-coil domain-containing protein 58	Q8R3Q6	CCDC58	1.907
31	Haloacid dehalogenase-like hydrolase domain-containing protein 3	Q9CYW4	HDHD3	0.602
32	tRNA methyltransferase 10 homolog C	Q3UFY8	TRMT10C	1.503
33	Deaminated glutathione amidase	Q8VDK1	NIT1	0.571
34	LETM1 domain-containing protein 1	Q924L1	LETMD1	0.656
35	Aurora kinase A-interacting protein	Q9DCJ7	AURKAIP1	1.610

**Table 2:** Primer sequences for qRT-PCR

Gene	Primer sequence
PDHE1α	Forward 5’-GGG ACG TCT GTT GAG AGA GC-3’
	Reverse 5’-TGT GTC CAT GGT AGC GGT AA-3’
PDK4	Forward 5’-CAG CTG GTG AAG AGC TGG TA-3’
	Reverse 5’-CTC TGA CAG GGC TTT CTG GT-3’

### Increased PDK4 and phospho-PDH is associated with markedly attenuated PDH activity in septic heart

**Fig. 3:**
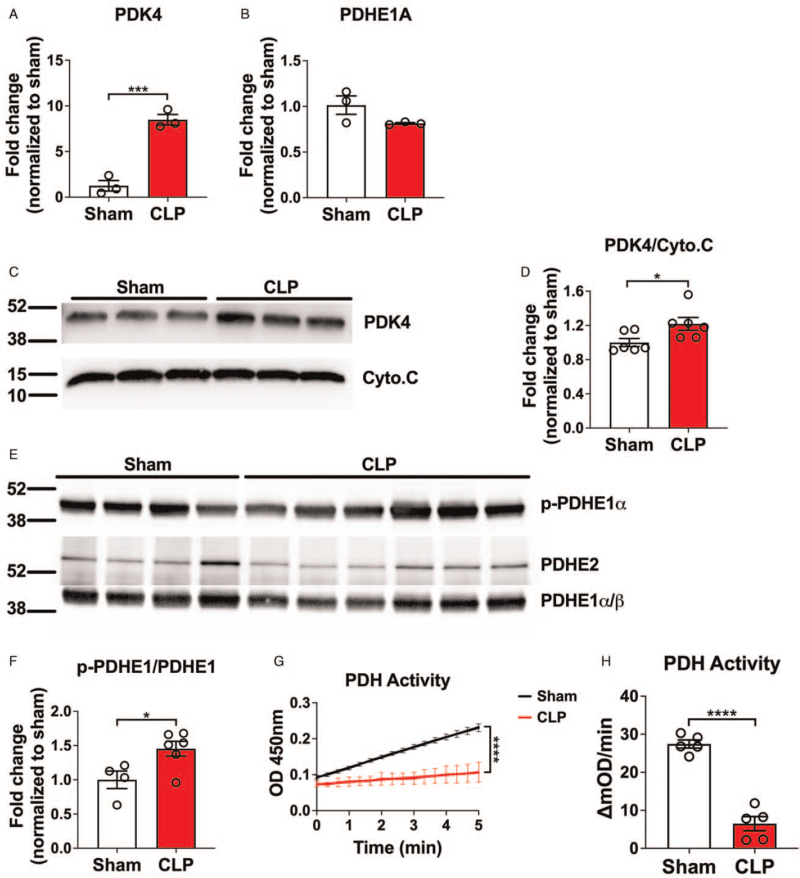
Functional and molecular analysis of PDH in septic hearts. **(**A and B) qRT-PCR analysis of myocardial PDK4 and PDHE1A gene expression, respectively, in sham or CLP mice. N = 3 mice/group. (C and D) Western blot of PDK4 in isolated mitochondria from sham and septic hearts. Densitometry analysis was done using Image J software and PDK4 levels were normalized to cytochrome C (Cyto.C). n = 6/group. (E) Western blot of serine 293-phosphorylated (p-PDHE1α), PDHE1α/β, and PDHE2 in whole hearts from sham and sepsis mice. PDH antibody detects two subunits of PDH, E2 and E1α/β. (F) Densitometry analysis was done using Image J software and p-PDHE1α levels were normalized to total PDHE1α/β levels. n = 4 (sham), 6 (CLP) mice. (G) Quantification of PDH activity over time. (H) Bar graph quantifying the change in PDH activity per min. PDH activity was calculated using the formula described in *Methods.* n = 5/group. Each error bar represents mean ± SEM. For all panels, data was analyzed using two-tailed *t* test. ^∗^*P* < 0.05, ^∗∗∗^*P* < 0.001, ^∗∗∗∗^*P* < 0.0001 (PDH, pyruvate dehydrogenase; PDHE1α/β, pyruvate dehydrogenase E1 subunit α and subunit β; PDHE1A, pyruvate dehydrogenase E1 subunit alpha; PDK4, pyruvate dehydrogenase kinase 4; p-PDHE1α, phosphorylated pyruvate dehydrogenase E1 subunit α).

We performed qRT-PCR to test whether the observed increase in mitochondrial PDK4 protein expression in septic hearts was due to increased transcription. We found a 6.8-fold increase in PDK4 gene expression in the cardiac tissue of septic mice as compared to sham mice (Fig. 3A, *P* < 0.001). In contrast, there was no difference in the gene expression of PDH E1α subunit between sham and sepsis hearts (Fig. 3B). In consistent with PDK4 gene expression, we also observed an increase in PDK4 protein expression in mitochondria isolated from septic hearts as compared with that of sham hearts (Fig. 3C and D, *P* < 0.05). Since the primary role of PDK4 is to phosphorylate the PDH E1 subunit, which is known to inhibit PDH enzyme activity, we performed Western blots using whole heart tissue from sham and CLP mice and found a significant increase in phosphorylated PDH at serine-293 in septic hearts (Fig. 3E and F). We then examined whether these molecular modifications were associated with altered PDH activities. We discovered that the myocardial tissues derived from septic mice had markedly lower PDH activity than that of sham mice (6.5 ± 4.2 vs. 27.4 ± 2.4 ΔmOD/min, Sham vs. CLP, n = 5, *P* < 0.0001) (Fig. 3G and H).

### Cardiac mitochondria of septic mice exhibited a reduction in ADP-stimulated and uncoupled oxygen consumption rate when fueled with pyruvate/malate

**Fig. 4:**
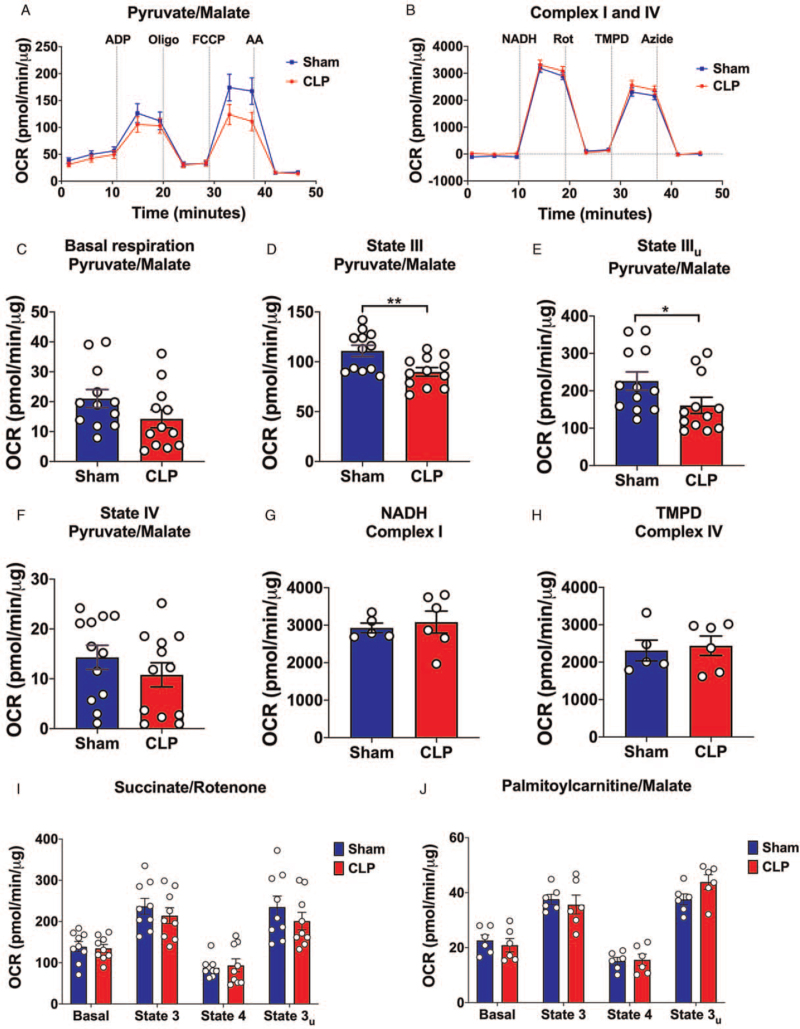
Oxygen consumption by cardiac mitochondria of sham and CLP mice. Heart mitochondria were isolated from sham or CLP mice 24 h after surgery. (A and B) Representative tracings of mitochondrial OCR in triplicate with pyruvate/malate as substrates (A) or subjected to the electron donor NADH-based assay to test Complex I and IV respiration (B). For the pyruvate/malate-fueled respiration assay, oxygen consumption was measured before and after the sequential additions of ADP, oligomycin, FCCP, and antimycin A. For the NADH-based assay, oxygen consumption was measured before and after the sequential additions of NADH+alamethicin+cytochrome C, rotenone, TMPD+ascorbate+antimycin A, and sodium azide. Protocol for all injections is described in the section of *Materials and Methods*. (C–F) OCR for sham and CLP mitochondria during basal (basal—antimycin A), state 3 (ADP—antimycin A), state 4 (oligomycin—antimycin A), and state 3 uncoupled (state 3_u,_ FCCP—antimycin A) respiration in the presence of pyruvate and malate. n = 12 mice/group. (G and H) OCR for sham and CLP mitochondria during Complex I (NADH—rotenone, G) and Complex IV (TMPD—azide, H)-dependent respiration. n *=* 5 (sham) and 6 (CLP). (I–J) OCR for sham CLP mitochondria during basal, state 3, state 4, and state 3 uncoupled in the presence of succinate and rotenone, or palmityolcarnitine and malate. n = 6 to 9 mice/group. Each dot represents respiration data from a single mouse mitochondrial preparation measured in technical replicates. Each error bar represents mean ± SEM. For (C and J), data was analyzed using two-tailed *t* test unless the data did not pass the normality test. In that case, data was analyzed by Mann–Whitney test. ^∗^*P* < 0.05, ^∗∗^*P* < 0.01. (AA, antimycin A; ADP, adenosine diphosphate; FCCP, trifluoromethoxy carbonylcyanide phenylhydrazone; OCR, oxygen consumption rate; oligo, oligomycin; Rot, rotenone; TMPD, N,N,N’,N’-tetramethyl-p-phenylenediamine).

We assessed mitochondrial oxygen consumption rate (OCR) using a Seahorse Extracellular Flux assay. To distinguish between pyruvate-driven respiration that depends on both PDH activity and Complex I and respiration that depends on Complex I only, isolated mitochondria were either fueled with pyruvate/malate as substrates or were permeabilized to directly deliver the Complex I substrate NADH. The exogenous electron donor TMPD was tested downstream of NADH addition to assess Complex IV activity (Fig. 4A and B). We found that compared to mitochondria from sham mice, mitochondria isolated from septic hearts exhibited a similar level of basal respiration (Fig. 4C), but significantly lower state 3 respiration (ADP-stimulated) (110.90 ± 5.65 vs. 89.79 ± 4.31 pmol O_2_/min/μg, sham vs. CLP, n = 12, *P* < 0.01) (Fig. 4D) and state 3 uncoupled respiration (226.20 ± 24.37 vs. 160.90 ± 21.57 pmol O_2_/min/μg, sham vs. CLP, n = 12, *P* < 0.05) (Fig. 4E) when fueled with pyruvate/malate. States IV respiration was unchanged (Fig. 4F). Importantly, NADH-fueled OCR (Complex I) and TMPD-fueled OCR (Complex IV) each exceeded the maximal pyruvate/malate-driven OCR by ≥10-fold and displayed no differences between sham and CLP (Fig. 4G and H). Moreover, succinate (in the presence of rotenone) and palmitoylcarnitine were also used as substrates to assess Complex II-dependent respiration and beta oxidation-driven respiration, respectively. No OCR differences between septic and sham mitochondria were observed when mitochondrial respirations were fueled by these substrates (Fig. 4I and J). The decreased OCR in the presence of pyruvate/malate but not Complex I, Complex II, Complex IV, or beta-oxidation substrates suggests that CLP mitochondria may have reduced capacity to metabolize pyruvate leading to the reduced stage III respiration. In addition, the dramatically lower uncoupled OCR by mitochondria oxidizing pyruvate/malate compared with Complex I (NADH) or IV (TMPO) substrates is consistent with the notion that NADH supply from PDH and other mitochondrial matrix dehydrogenase enzymes is rate-limiting for mitochondrial respiration in the heart.

## DISCUSSION

In a mouse model of polymicrobial sepsis, we demonstrated sepsis-induced cardiomyopathy with significant contractile dysfunction as illustrated by echocardiographic measurements *in vivo* and in isolated Langendorff-perfused hearts *ex vivo*. Energy production and/or demand may also be reduced as septic hearts exhibited a higher ADP/ATP ratio. To profile mitochondrial proteins in the heart, we carried out a quantitative proteomics analysis on cardiac mitochondria purified from septic and sham mice, which revealed differential expressions of 35 mitochondrial proteins between sepsis and sham mice. One of the mitochondrial proteins, PDK4, the primary kinase that phosphorylates and inactivates PDH, was upregulated whereas PDH activity was markedly decreased in the septic hearts. Moreover, oxygen consumption rate (OCR) by mitochondria from the septic hearts were significantly decreased as compared with the sham controls when respiration was fueled by pyruvate and malate. Importantly, OCR did not differ between the two groups when NADH and TMPD were used to directly drive Complex I and IV respiration, respectively, or when succinate was used to test respiration that is dependent on Complex II and downstream components of the ETC, or when the beta-oxidation substrate palmitoylcarnitine was used. These data suggest that in murine sepsis, cardiac dysfunction is associated with marked mitochondrial PDH inhibition and impaired pyruvate-fueled oxidative respiration in the heart.

The proteomic analysis identified a total of 1,135 proteins, 665 of which appeared to be mitochondria or metabolism-associated proteins. To enhance rigor in our study, we only focused on proteins that were highly abundant in both the sham and the CLP groups. Of these proteins, we focused on mitochondrial proteins that increase/decrease by approximately ∼1.5 fold with such effects having statistical significance of a *P* < 0.05. Furthermore, we would like to stress that no conclusion was made solely based on the proteomic data. Any protein of interest highlighted by the proteomic analysis was further tested by another method. It should be noted that while proteins belonging to all four ETC complexes were detected in both sham and CLP cardiac mitochondria, only Complex I and Complex IV subunits exhibited differential expression between sham and CLP mice. In addition, we found other proteins involved in pyruvate metabolism, fatty acid metabolism, and electron transport to be perturbed in mitochondria isolated from the septic hearts. Notably, enzymes traditionally involved in beta-oxidation did not show altered expression. Therefore, the functional significance of the proteomic findings of these enzymes, that is, methylmalonyl-CoA epimerase (MCE), phytanol-CoA dioxygenase (PHYH), and carbonyl reductase [NADPH] 2 (CBR2) remains to be investigated. MCE catabolizes fatty acids with odd-length carbon chains. PHYH is a peroxisome-associated protein involved in alpha-oxidation of fatty acids, and CBR2 metabolizes aldehydes and ketones derived from lipid peroxidation (35). Lipid peroxidation occurs when free radicals gain electrons from lipids, causing the formation of fatty acid radicals. As sepsis is known to generate high levels of reactive oxygen species (ROS) (36, 37), it is speculated that these mitochondria have fatty acid radicals, resulting in an increased need for CBR2-dependent detoxification. Moreover, we did not see reduction in the adenine nucleotide translocator (ANT1) in cardiac mitochondria of CLP mice, in contrast to a report that TNFα-treated mice have myocardial inflammation and reduced ANT1 expression (16). Overall, the proteomics analysis revealed several metabolic pathways that were disturbed in septic mitochondria, providing the molecular basis for future explorations of the mechanisms of mitochondrial dysfunction in sepsis-induced cardiomyopathy.

One of the interesting findings in this study was the phosphorylation and inhibition of cardiac PDH activity during sepsis. PDH activity is an overall rate-limiting step in oxidative phosphorylation that is driven by pyruvate. Therefore, the reduction in OCR driven by pyruvate/malate may be explained by the observed PDK4 upregulation and PDH inhibition in septic heart. It should be pointed out that similar change in PDH activity has been reported in other cells/tissues in sepsis, such as skeletal muscle, peripheral blood mononuclear cells (38–42), and splenocytes/hepatocytes (18). Moreover, McCall and co-workers reported that inhibition of PDK using dichloroacetate reversed PDH phosphorylation and improved mitochondrial respiration in hepatocytes and splenocytes (18). Using a chronic sepsis model induced by intra-abdominal placement of a fecal-agar pellet containing *E. coli* and *B. fragilis* in rats, Vary and co-workers demonstrated PDH inactivation in skeletal muscle after 5 days. Others demonstrate PDK4 upregulation and PDH inhibition following LPS treatment in cells and in animal models (43, 44). In a swine model, LPS infusion led to elevated levels of PDK4 transcript in the myocardium (45), although PDH activity was not assessed in the study. Expanding from these early studies, we identified a functional association between PDH inactivation and significant and specific reduction in pyruvate/malate-fueled OCR in the heart and in the absence of Complex I, II, and VI dysfunction. Moreover, and somewhat surprisingly, we did not find any difference between sham and septic mitochondria in the fatty acid palmitoylcarnitine-fueled mitochondrial respiration.

Currently, there are few therapies specifically designed to target myocardial bioenergetics in SIC. The standard clinical management of sepsis and septic shock includes antibiotics, fluid resuscitation, vasopressors, and positive inotropes among others (46–48). Other investigative therapies include preventative approaches such as the use of 3-hydroxy-3-methylglutaryl coenzyme A reductase or statins, which have anti-inflammatory and antioxidative properties. Statins in particular, have been shown to considerably reduce the rate of severe sepsis and ICU admissions although there were no differences in mortality (47). Based on our data and the data from previous studies, we speculate that inhibition of PDK may promote PDH activity and pyruvate-driven oxidative phosphorylation, improve mitochondrial bioenergetic in septic heart, and thus represent a novel therapeutic approach for sepsis-induced cardiomyopathy.

Surprisingly, despite the proteomics data demonstrating a significant decrease in NADH dehydrogenase 1 beta subcomplex subunit 8 (NDUFB8), a Complex I accessory subunit, we did not find any functional impairment with the NADH-driven Complex I respiration in mitochondria isolated from the septic hearts. Several previous studies suggested that Complex I activity is reduced during sepsis or endotoxemia in the heart and the brain, including studies that utilized isolated cardiac mitochondria (15, 49, 50). However, these studies reported decreased enzymatic activity of Complex I, but did not assess the functional ability of the mitochondria to utilize Complex I-linked substrates that depend on the activity of the citric acid cycle dehydrogenases such as PDH. Another study into the role of Parkin during endotoxemia did investigate mitochondrial function in the heart after single-dose intraperitoneal injection of either PBS or LPS in WT or *park2*^*−/−*^ mice (51). This study found reduced Complex I activity in the hearts of WT mice 12 h after LPS injection, which recovered at 48 h post-LPS treatment.

There were several limitations in the study. For one, we did not verify the changes of all 35 of the differentially expressed proteins that were found in our proteomics data by Western blot, as we elected to focus more on PDH and mitochondrial oxidative phosphorylation function. We also did not test all of the differentially expressed proteins found by the proteomics and there were likely some changes to low abundance proteins that were below the sensitivity of detection. Therefore, there are probably additional metabolically related proteins that are involved in the cardiac energy deficit during sepsis. It must be stressed that molecular remodeling, as identified by mass spec, even when confirmed by other molecular tests such as Western blot, does not necessarily imply functional impairment. Additionally, substrate availability in the mitochondrial respiration experiments is unlimited whereas bioavailability *in vivo* during sepsis may be compromised, especially with a decrease in energy demand in the septic heart, for example, slower heart rate and lower core temperature. More comprehensive functional testing of the metabolic pathways identified by the proteomics data will be pursued in our future studies.

**Fig. 5:**
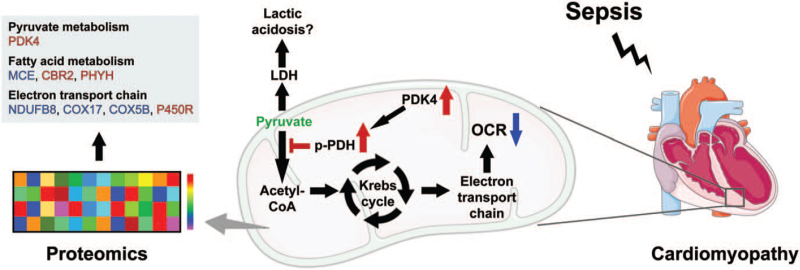
Molecular remodeling of cardiac mitochondrial in murine sepsis. Proteomic analysis of mitochondria from hearts with sepsis-induced cardiomyopathy revealed several mitochondria-specific proteins with altered expression including PDK4 and the electron transport chain proteins NDUFB8, COX17, COX5B, and P450R. Increase in PDK4 expression leads to phosphorylation and inactivation of PDH, the enzyme converting pyruvate, NAD^+^, and coenzyme A into acetyl-CoA, CO_2_, and NADH. Diminished PDH activity limits the overall capacity of the mitochondria to utilize the metabolic substrate pyruvate resulting in a lower oxygen consumption rate as shown by the schematic above. These molecular modifications may provide insight into how the mitochondria is linked to the development of sepsis-induced cardiomyopathy. (CBR2, carbonyl reductase [NADPH] 2; COX 17, cytochrome c oxidase copper chaperone; COX5B, cytochrome c oxidase 5B; LDH, lactate dehydrogenase; MCE, methylmalonyl-CoA epimerase; NDUFB8, NADH ubiquinone oxioreductase subunit B8; OCR, oxygen consumption rate; P450R, NADPH-cytochrome P450 reductase; PDK4, pyruvate dehydrogenase kinase 4; PHYH, Phytanoyl-CoA 2-Hydroxylase; p-PDH, phospho-pyruvate dehydrogenase).

In summary, by employing a proteomics approach, we identified 35 mitochondrial or metabolism-associated proteins that displayed differential expression in cardiac mitochondria from mice with SIC when compared with sham control (Fig. 5). Notably, PDK4 was upregulated at both mRNA and protein levels, along with an increase in the phosphorylated PDH and marked reduction in PDH activity in septic heart homogenate. These molecular remodeling events were associated with reduced mitochondrial capability to utilize pyruvate and likely contributed to decreased oxidative phosphorylation in the septic heart. These findings provide a molecular framework to better understanding the etiology of sepsis and sepsis-induced cardiomyopathy.

## Supplementary Material

Supplemental Digital Content

## References

[1] Cimolai MC Alvarez S Bode C Bugger H. Mitochondrial mechanisms in septic cardiomyopathy. *Int J Mol Sci* 16 (8):17763–17778, 2015. 26247933 10.3390/ijms160817763 PMC4581220

[2] Court O Kumar A Parrillo JE Kumar A. Clinical review: myocardial depression in sepsis and septic shock. *Crit Care* 6 (6):500–508, 2002. 12493071 10.1186/cc1822 PMC153435

[3] Stanzani G Duchen MR Singer M. The role of mitochondria in sepsis-induced cardiomyopathy. *Biochim Biophys Acta Mol Basis Dis* 1865 (4):759–773, 2019. 30342158 10.1016/j.bbadis.2018.10.011

[4] Wilhelm J Hettwer S Schuermann M Bagger S Gerhardt F Mundt S Muschik S Zimmermann J Bubel S Amoury M. Severity of cardiac impairment in the early stage of community-acquired sepsis determines worse prognosis. *Clin Res Cardiol* 102 (10):735–744, 2013. 23740197 10.1007/s00392-013-0584-z

[5] Levy RJ. Mitochondrial dysfunction, bioenergetic impairment, and metabolic down-regulation in sepsis. *Shock* 28 (1):24–28, 2007. 17483747 10.1097/01.shk.0000235089.30550.2d

[6] Brealey D Brand M Hargreaves I Heales S Land J Smolenski R Davies NA Cooper CE Singer M. Association between mitochondrial dysfunction and severity and outcome of septic shock. *Lancet* 360 (9328):219–223, 2002. 12133657 10.1016/S0140-6736(02)09459-X

[7] Barth E Stammler G Speiser B Schaper J. Ultrastructural quantitation of mitochondria and myofilaments in cardiac muscle from 10 different animal species including man. *J Mol Cell Cardiol* 24 (7):669–681, 1992. 1404407 10.1016/0022-2828(92)93381-s

[8] Briese V Than G Richter D Iino K Seppala M. Placental protein 5 and pregnancy zone protein in ovarian cysts and benign cystic ovarian tumors. *Zentralbl Gynakol* 110 (13):821–823, 1988. 3176735

[9] Brown DA Perry JB Allen ME Sabbah HN Stauffer BL Shaikh SR Cleland JG Colucci WS Butler J Voors AA. Expert consensus document: mitochondrial function as a therapeutic target in heart failure. *Nat Rev Cardiol* 14 (4):238–250, 2017. 28004807 10.1038/nrcardio.2016.203 PMC5350035

[10] Wescott AP Kao JPY Lederer WJ Boyman L. Voltage-energized calcium-sensitive ATP production by mitochondria. *Nat Metab* 1 (10):975–984, 2019. 31950102 10.1038/s42255-019-0126-8 PMC6964030

[11] Nicholls DG Ferguson SJ. Bioenergetics. Amsterdam: Academic Press, Elsevier; 2013.

[12] Chopra M Golden HB Mullapudi S Dowhan W Dostal DE Sharma AC. Modulation of myocardial mitochondrial mechanisms during severe polymicrobial sepsis in the rat. *PLoS One* 6 (6):e21285, 2011. 21712982 10.1371/journal.pone.0021285 PMC3119671

[13] Reynolds CM Suliman HB Hollingsworth JW Welty-Wolf KE Carraway MS Piantadosi CA. Nitric oxide synthase-2 induction optimizes cardiac mitochondrial biogenesis after endotoxemia. *Free Radic Biol Med* 46 (5):564–572, 2009. 19073249 10.1016/j.freeradbiomed.2008.11.007 PMC2666005

[14] Kozlov AV Staniek K Haindl S Piskernik C Ohlinger W Gille L Nohl H Bahrami S Redl H. Different effects of endotoxic shock on the respiratory function of liver and heart mitochondria in rats. *Am J Physiol Gastrointest Liver Physiol* 290 (3):G543–549, 2006. 16474010 10.1152/ajpgi.00331.2005

[15] Vanasco V Saez T Magnani ND Pereyra L Marchini T Corach A Vaccaro MI Corach D Evelson P Alvarez S. Cardiac mitochondrial biogenesis in endotoxemia is not accompanied by mitochondrial function recovery. *Free Radic Biol Med* 77:1–9, 2014. 25224040 10.1016/j.freeradbiomed.2014.08.009

[16] Pan S Wang N Bisetto S Yi B Sheu SS. Downregulation of adenine nucleotide translocator 1 exacerbates tumor necrosis factor-alpha-mediated cardiac inflammatory responses. *Am J Physiol Heart Circ Physiol* 308 (1):H39–48, 2015. 25380814 10.1152/ajpheart.00330.2014 PMC4281676

[17] Dawson KL Geller ER Kirkpatrick JR. Enhancement of mitochondrial function in sepsis. *Arch Surg* 123 (2):241–244, 1988. 3277586 10.1001/archsurg.1988.01400260129017

[18] McCall CE Zabalawi M Liu T Martin A Long DL Buechler NL Arts RJW Netea M Yoza BK Stacpoole PW. Pyruvate dehydrogenase complex stimulation promotes immunometabolic homeostasis and sepsis survival. *JCI Insight* 3 (15):e99292, 2018. 10.1172/jci.insight.99292 PMC6129136 30089711

[19] Bowker-Kinley MM Davis WI Wu P Harris RA Popov KM. Evidence for existence of tissue-specific regulation of the mammalian pyruvate dehydrogenase complex. *Biochem J* 329 (Pt 1):191–196, 1998. 9405293 10.1042/bj3290191 PMC1219031

[20] Korotchkina LG Patel MS. Site specificity of four pyruvate dehydrogenase kinase isoenzymes toward the three phosphorylation sites of human pyruvate dehydrogenase. *J Biol Chem* 276 (40):37223–37229, 2001. 11486000 10.1074/jbc.M103069200

[21] Zou L Feng Y Chen YJ Si R Shen S Zhou Q Ichinose F Scherrer-Crosbie M Chao W. Toll-like receptor 2 plays a critical role in cardiac dysfunction during polymicrobial sepsis. *Crit Care Med* 38 (5):1335–1342, 2010. 20228680 10.1097/CCM.0b013e3181d99e67 PMC3997231

[22] Rittirsch D Huber-Lang MS Flierl MA Ward PA. Immunodesign of experimental sepsis by cecal ligation and puncture. *Nat Protoc* 4 (1):31–36, 2009. 19131954 10.1038/nprot.2008.214 PMC2754226

[23] Zou L Feng Y Zhang M Li Y Chao W. Nonhematopoietic toll-like receptor 2 contributes to neutrophil and cardiac function impairment during polymicrobial sepsis. *Shock* 36 (4):370–380, 2011. 21701420 10.1097/SHK.0b013e3182279868 PMC3178725

[24] Zou L Feng Y Li Y Zhang M Chen C Cai J Gong Y Wang L Thurman JM Wu X. Complement factor B is the downstream effector of TLRs and plays an important role in a mouse model of severe sepsis. *J Immunol* 191 (11):5625–5635, 2013. 24154627 10.4049/jimmunol.1301903 PMC3906719

[25] Rogers GW Brand MD Petrosyan S Ashok D Elorza AA Ferrick DA Murphy AN. High throughput microplate respiratory measurements using minimal quantities of isolated mitochondria. *PLoS One* 6 (7):e21746, 2011. 21799747 10.1371/journal.pone.0021746 PMC3143121

[26] Acin-Perez R Benador IY Petcherski A Veliova M Benavides GA Lagarrigue S Caudal A Vergnes L Murphy AN Karamanlidis G. A novel approach to measure mitochondrial respiration in frozen biological samples. *EMBO J* 39 (13):e104073, 2020. 32432379 10.15252/embj.2019104073 PMC7327496

[27] Bordt EA Clerc P Roelofs BA Saladino AJ Tretter L Adam-Vizi V Cherok E Khalil A Yadava N Ge SX. The putative Drp1 inhibitor mdivi-1 is a reversible mitochondrial complex I inhibitor that modulates reactive oxygen species. *Dev Cell* 40 (6):583–594.e6, 2017. 28350990 10.1016/j.devcel.2017.02.020 PMC5398851

[28] Jaber SM Yadava N Polster BM. Mapping mitochondrial respiratory chain deficiencies by respirometry: beyond the mito stress test. *Exp Neurol* 328:113282, 2020. 32165258 10.1016/j.expneurol.2020.113282 PMC7202675

[29] Wisniewski JR Zougman A Nagaraj N Mann M. Universal sample preparation method for proteome analysis. *Nat Methods* 6 (5):359–362, 2009. 19377485 10.1038/nmeth.1322

[30] Erde J Loo RR Loo JA. Enhanced FASP (eFASP) to increase proteome coverage and sample recovery for quantitative proteomic experiments. *J Proteome Res* 13 (4):1885–1895, 2014. 24552128 10.1021/pr4010019 PMC3993969

[31] Williamson JC Edwards AV Verano-Braga T Schwammle V Kjeldsen F Jensen ON Larsen MR. High-performance hybrid Orbitrap mass spectrometers for quantitative proteome analysis: observations and implications. *Proteomics* 16 (6):907–914, 2016. 26791339 10.1002/pmic.201400545

[32] Eng JK Fischer B Grossmann J Maccoss MJ. A fast SEQUEST cross correlation algorithm. *J Proteome Res* 7 (10):4598–4602, 2008. 18774840 10.1021/pr800420s

[33] Dorfer V Pichler P Stranzl T Stadlmann J Taus T Winkler S Mechtler K. MS Amanda, a universal identification algorithm optimized for high accuracy tandem mass spectra. *J Proteome Res* 13 (8):3679–3684, 2014. 24909410 10.1021/pr500202e PMC4119474

[34] Kall L Canterbury JD Weston J Noble WS MacCoss MJ. Semi-supervised learning for peptide identification from shotgun proteomics datasets. *Nat Methods* 4 (11):923–925, 2007. 17952086 10.1038/nmeth1113

[35] Nakanishi M Deyashiki Y Ohshima K Hara A. Cloning, expression and tissue distribution of mouse tetrameric carbonyl reductase. Identity with an adipocyte 27-kDa protein. *Eur J Biochem* 228 (2):381–387, 1995. 7705352

[36] Zang Q Maass DL Tsai SJ Horton JW. Cardiac mitochondrial damage and inflammation responses in sepsis. *Surg Infect (Larchmt)* 8 (1):41–54, 2007. 17381396 10.1089/sur.2006.033 PMC6044285

[37] Yao X Carlson D Sun Y Ma L Wolf SE Minei JP Zang QS. Mitochondrial ROS induces cardiac inflammation via a pathway through mtDNA damage in a pneumonia-related sepsis model. *PLoS One* 10 (10):e0139416, 2015. 26448624 10.1371/journal.pone.0139416 PMC4598156

[38] Vary TC Hazen S. Sepsis alters pyruvate dehydrogenase kinase activity in skeletal muscle. *Mol Cell Biochem* 198 (1–2):113–118, 1999. 10497885 10.1023/a:1006993910781

[39] Nuzzo E Berg KM Andersen LW Balkema J Montissol S Cocchi MN Liu X Donnino MW. Pyruvate dehydrogenase activity is decreased in the peripheral blood mononuclear cells of patients with sepsis. A prospective observational trial. *Ann Am Thorac Soc* 12 (11):1662–1666, 2015. 26356483 10.1513/AnnalsATS.201505-267BC PMC4724894

[40] Vary TC. Sepsis-induced alterations in pyruvate dehydrogenase complex activity in rat skeletal muscle: effects on plasma lactate. *Shock* 6 (2):89–94, 1996. 8856841 10.1097/00024382-199608000-00002

[41] Vary TC Martin LF. Potentiation of decreased pyruvate dehydrogenase activity by inflammatory stimuli in sepsis. *Circ Shock* 39 (4):299–305, 1993. 8485821

[42] Alamdari N Constantin-Teodosiu D Murton AJ Gardiner SM Bennett T Layfield R Greenhaff PL. Temporal changes in the involvement of pyruvate dehydrogenase complex in muscle lactate accumulation during lipopolysaccharide infusion in rats. *J Physiol* 586 (6):1767–1775, 2008. 18218678 10.1113/jphysiol.2007.149625 PMC2375698

[43] Park H Jeoung NH. Inflammation increases pyruvate dehydrogenase kinase 4 (PDK4) expression via the Jun N-terminal kinase (JNK) pathway in C2C12 cells. *Biochem Biophys Res Commun* 469 (4):1049–1054, 2016. 26740179 10.1016/j.bbrc.2015.12.113

[44] Thomas GW Mains CW Slone DS Craun ML Bar-Or D. Potential dysregulation of the pyruvate dehydrogenase complex by bacterial toxins and insulin. *J Trauma* 67 (3):628–633, 2009. 19741411 10.1097/TA.0b013e3181a8b415

[45] Lado-Abeal J Martinez-Sanchez N Cocho JA Martin-Pastor M Castro-Piedras I Couce-Pico ML Saha AK Lopez M. Lipopolysaccharide (LPS)-induced septic shock causes profound changes in myocardial energy metabolites in pigs. *Metabolomics* 14 (10):131, 2018. 30830414 10.1007/s11306-018-1433-x

[46] Kakihana Y Ito T Nakahara M Yamaguchi K Yasuda T. Sepsis-induced myocardial dysfunction: pathophysiology and management. *J Intensive Care* 4:22, 2016. 27011791 10.1186/s40560-016-0148-1 PMC4804632

[47] Merx MW Weber C. Sepsis and the heart. *Circulation* 116 (7):793–802, 2007. 17698745 10.1161/CIRCULATIONAHA.106.678359

[48] Celes MR Prado CM Rossi MA. Sepsis: going to the heart of the matter. *Pathobiology* 80 (2):70–86, 2013. 22986917 10.1159/000341640

[49] Comim CM Rezin GT Scaini G Di-Pietro PB Cardoso MR Petronilho FC Ritter C Streck EL Quevedo J Dal-Pizzol F. Mitochondrial respiratory chain and creatine kinase activities in rat brain after sepsis induced by cecal ligation and perforation. *Mitochondrion* 8 (4):313–318, 2008. 18657632 10.1016/j.mito.2008.07.002

[50] Zang QS Martinez B Yao X Maass DL Ma L Wolf SE Minei JP. Sepsis-induced cardiac mitochondrial dysfunction involves altered mitochondrial-localization of tyrosine kinase Src and tyrosine phosphatase SHP2. *PLoS One* 7 (8):e43424, 2012. 22952679 10.1371/journal.pone.0043424 PMC3428365

[51] Piquereau J Godin R Deschenes S Bessi VL Mofarrahi M Hussain SN Burelle Y. Protective role of PARK2/Parkin in sepsis-induced cardiac contractile and mitochondrial dysfunction. *Autophagy* 9 (11):1837–1851, 2013. 24121678 10.4161/auto.26502

