# Supplementary material for: Pyruvate-Driven Oxidative Phosphorylation is Downregulated in Sepsis-Induced Cardiomyopathy: A Study of Mitochondrial Proteome
Source: Shock. 2021 Sep 9;57(4):553–64. doi: 10.1097/SHK.0000000000001858 (PMC8904652; doi:10.1097/SHK.0000000000001858)
Supplement: Supplemental Digital Content [file shk-57-553-s001.docx]

**SUPPLEMENTAL FIGURE**

**Pyruvate-driven Oxidative Phosphorylation Is Downregulated in Sepsis-induced Cardiomyopathy: A Study of Mitochondrial Proteome**

Briana K. Shimada, PhD^1^, Liron Boyman, PhD^2^, Weiliang Huang, PhD^3^, Jing Zhu, MD, PhD^1^, Yang Yang, MD^1^, Fengqian Chen, PhD^1^, Maureen A. Kane, PhD^3^, Nagendra Yadava, PhD^1^, Lin Zou, MD, PhD^1^, W. Jonathan Lederer, MD, PhD^2^, Brian M. Polster, PhD^1^, and

Wei Chao, MD, PhD^1^

**Short Title: Mitochondrial proteomics in septic heart**

^1^ Translational Research Program, Department of Anesthesiology & Center for Shock, Trauma and Anesthesiology Research, Baltimore, MD, USA

^2^ The Department of Physiology and Center for Biomedical Engineering and Technology, University of Maryland School of Medicine, Baltimore, MD, USA

^3^ Department of Pharmaceutical Sciences, University of Maryland School of Pharmacy, Baltimore, MD, USA

**Supplemental Figure 1**

**
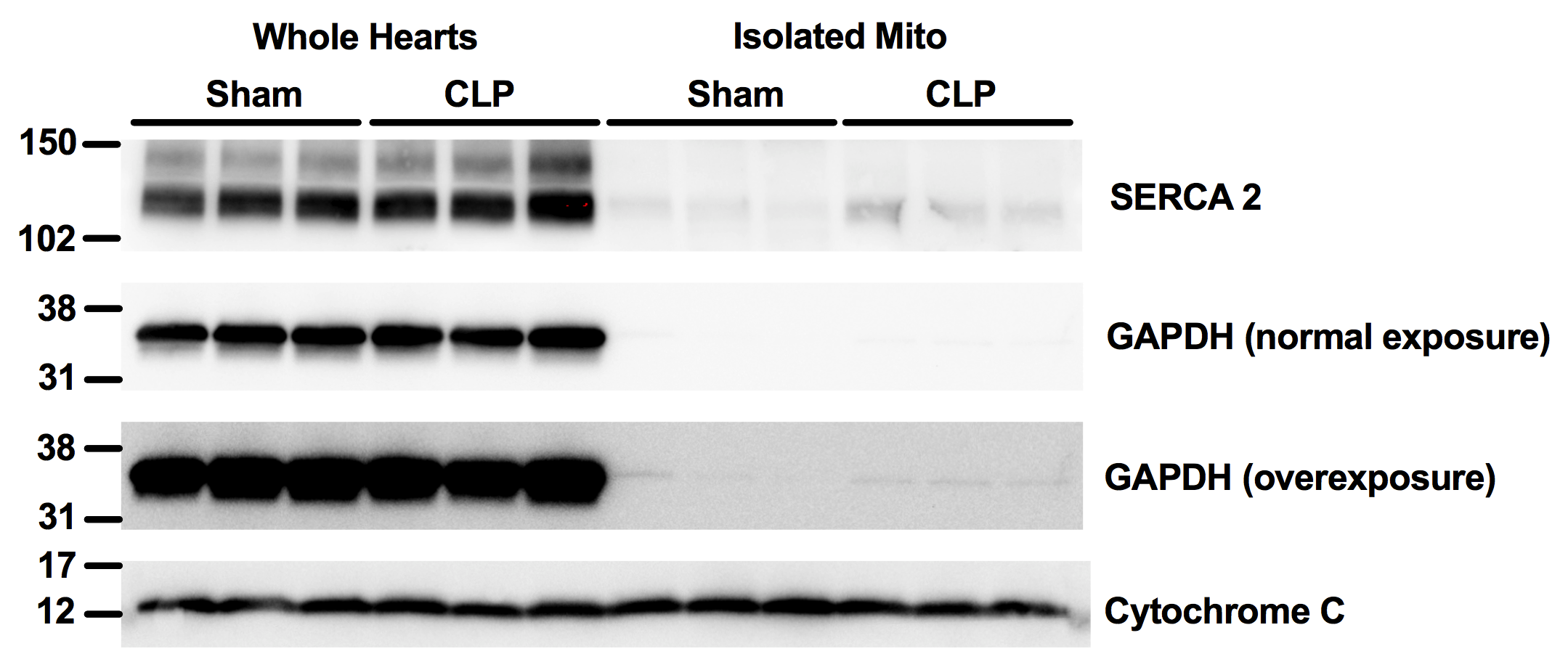
**

**Supplemental Figure 1. Assessment of non-mitochondrial proteins in isolated mitochondria.** Western blots of GAPDH, SERCA, and cytochrome c in whole heart and isolated mitochondria from sham and CLP mice. GAPDH is shown using normal exposure and prolonged exposure. (Abbreviations: GAPDH, glyceraldehyde 3-phosphate dehydrogenase, SERCA, SR Ca^2+^-ATPase).
